# Understanding the Sidewall Passivation Effects in AlGaInP/GaInP Micro-LED

**DOI:** 10.1186/s11671-022-03669-5

**Published:** 2022-03-01

**Authors:** Juhyuk Park, Woojin Baek, Dae-Myeong Geum, Sanghyeon Kim

**Affiliations:** 1 School of Electrical Engineering, Korea Advanced Institute of Science and Technology (KAIST), Daejeon, 34141 Republic of Korea; 2 Infromation and Electronics Research Institute, Korea Advanced Institute of Science and Technology (KAIST), Daejeon, 34141 Republic of Korea

**Keywords:** Micro-LED, Passivation, AlGaInP/GaInP MQW

## Abstract

The passivation effects of sulfur treatment and Al_2_O_3_ passivation for AlGaInP/GaInP red micro-light-emitting-diodes (LEDs) were investigated in terms of the external quantum efficiency (EQE) and the current density showing the peak EQE (*J*_EQE, peak_). We systematically compared the electrical and optical characteristics of the micro-LEDs with and without passivation according to various sizes. Interestingly, our investigation indicated that simple electrical characteristics such as current density–voltage property are difficult to precisely reflect the minor change in electrical properties due to passivation when the device has the inherently low leakage current. Whereas the EQE was enhanced by 20% and *J*_EQE, peak_ was largely shifted to a lower current density region at the LED with a size of 15 × 15 μm^2^. To examine the passivation effects, we carefully analyzed the EQE and *J*_EQE, peak_ with the ABC recombination model, and established the methodology to investigate the influence of a sidewall in micro-LEDs. As a result, we extracted the surface recombination velocity regarding the surface passivation, showing a nearly 14% reduction with the passivation.

**Supplementary Information:**

The online version contains supplementary material available at 10.1186/s11671-022-03669-5.

## Introduction

Inorganic micro-light-emitting-diodes (LEDs) are in the spotlight for ultra-high resolution display devices due to its high performance such as high brightness, scalability, and contrast ratio [1, 2]. In order to implement the energy-efficient micro-LED display, high external quantum efficiency (EQE) should be achieved with a scaled pixel smaller than 50 × 50 μm^2^ which is a 1/100 size of a conventional LED [3]. Furthermore, a maximum EQE should be positioned at the relatively low injection current regime, which could lead to a strong benefit of a power consumption for micro-display compared with the LED for general lighting [4, 5]. However, the sidewall defects, which are mainly caused by the mesa formation process such as inductively-coupled plasma reactive ion etching (ICP-RIE), play a critical role as a Shockley–Read–Hall (SRH) recombination center. It results in the degradation of the EQE value especially for micro-LEDs because the smaller LEDs have a large surface-to-volume ratio as the device size decreases. Also it could cause the current density showing the maximum EQE (*J*_EQE, peak_) shift to a higher current density region [6, 7].

Therefore, to suppress the sidewall induced performance degradations, the various passivation strategies have been studied for inorganic micro-LEDs. For instance, the KOH treatment followed by an atomic layer deposition system (ALD) has been reported to passivate the InGaN micro-LED. With passivation, the light emission uniformity was improved, the EQE reduction by shrinking micro-LED dimensions was effectively reduced [8]. In the case of the AlGaInP/GaInP multi-quantum well (MQW) based red micro-LEDs, it is known to have a larger surface recombination velocity (SRV) than InGaN/GaN MQW, resulting in significant performance degradation with a pixel size scaling [9]. However, there are still few researches on the AlGaInP/GaInP passivation, although red LED is one of the important building blocks for fabricating future full-color micro-displays [10, 11]. Furthermore, most of the reported micro-LEDs showed a high leakage current, indicating the fabrication process has not much been optimized than InGaN/GaN LED. On the other hand, the investigation of EQE relative to injection current density has not been conducted yet in AlGaInP/GaInP MQW micro-LEDs because a low current injection regime would not be critically considered for lighting applications. Therefore, to fabricate the highly efficient micro-displays, the exploration of the fabrication technology of AlGaInP/GaInP LED including pixel formation process as well as surface passivation should be carried out. In addition, the investigation of EQE relative to injection current density including *J*_EQE, peak_ should be conducted to develop the red-pixels of display application and understand the effect of sidewall degradation.

In the present work, we fabricated the AlGaInP/GaInP red micro-LEDs and systematically investigated the EQE with various device sizes from 15 × 15 μm^2^ to 80 × 80 μm^2^. Furthermore, to investigate the passivation effect for AlGaInP/GaInP micro-LEDs, we conducted the conventional surface passivation with sulfur treatment followed by Al_2_O_3_ deposition [12–14]. Furthermore, the effect of the passivation was carefully examined in terms of the EQE enhancement and *J*_EQE, peak_ shift through optical and electrical characterizations. Then, to quantitively extract the SRV values, by fitting the measured EQE data with the ABC model of recombinations, the SRH recombination constants were well analyzed with respect to the micro-LED sizes.

## Methods

### Fabrication Process of Micro-LEDs

**Fig. 1:**
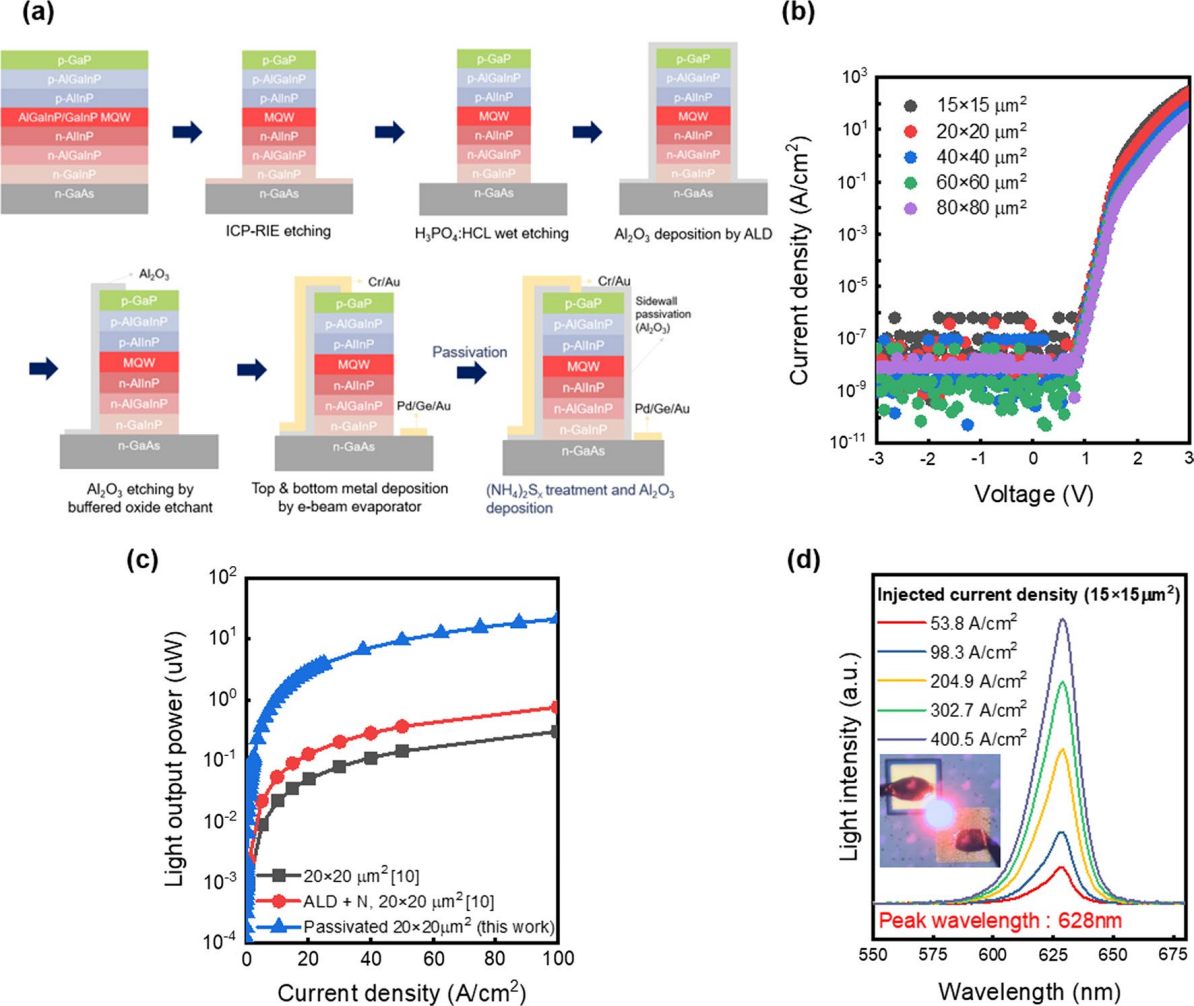
**a** The process flow of AlGaInP/GaInP MQW LED with passivation. **b** The current density–voltage (*J*–*V*) characteristic of AlGaInP/GaInP MQW LED depending on device sizes. **c** The current density–light output power (*J*–*L*) characteristics of 20 × 20 μm^2^ micro-LED comparing with the same size devices of Ref [10] **d** Electroluminescence of AlGaInP/GaInP MQW LED with different current density. The inset in (**d**) is the microscope image of 20 × 20 μm^2^ AlGaInP/GaInP MQW LED at 100 A/cm^2^

The epitaxial layers were grown by metal–organic chemical vapor deposition (MOCVD) on a 4-inch semi-insulating GaAs substrate. The micro-LED structure based on AlGaInP/GaInP MQW structure is shown in Fig. 1a. The micro-LED fabrication process was started with the standard cleaning process with acetone, methanol, and deionized (DI) water. Sequentially, the mesa isolation was conducted to define the pixel by ICP-RIE. We etched the AlGaInP/GaInP epilayers with Cl_2_ and Ar gas flow in an ICP-RIE system. In this step, the etching was carried out until the middle of the *n*-GaInP layer. Then, the samples were etched to completely remove the remaining *n*-GaInP layers and expose the *n*^+^-GaAs contact layer using the H_3_PO_4_: HCl (3:1) solution. For preventing electrical short problem of *p*-contact metal, Al_2_O_3_ layer was patially defined for *p*-conact region. After that, Cr/Au (25/75 nm) was deposited for *p*-type contact metal to GaP by the electron beam (e-beam) evaporator. Also, Pd/Ge/Au (20/40/100 nm) was deposited on the *n*^+^-GaAs layer. Finally, the sample was annealed at 200 °C for 10 min to reduce the contact resistance of ohmic contacts.

After the standard device fabrication, we carried out the surface passivation. The micro-LEDs were dipped in HCl: DI (1:5) solution for 30 s to remove the native oxide of the sidewall. Then, the sample was dipped in (NH_4_)_2_S_*x*_:DI (1:10) solution for 10 min and was subsequently loaded into the atomic layer deposition (ALD) chamber to minimize the naturally formed native oxide. Finally, after depositing the 10 nm Al_2_O_3_ passivation layer, the passivated micro-LEDs were analyzed comparatively with non-passivated micro-LED. The electrical characteristics were measured by Keithley 4200. For measuring optical characteristics, we firstly used the integrated sphere system. However, when using the integrated sphere, inevitable photon loss occurred since the micro-LED is located a few inches away from the inner surface of the sphere. Therefore, to measure the lower current density region, we used the photodetector located directly above the micro-LED and calibrated the measured photocurrent to light output power (W) by referring to the integrated sphere data (Additional file 1: Figure S1).

## Results and Discussion

### Electrical and Optical Characterization of the Fabricated Micro-LEDs

The electrical characteristics of the AlGaInP/GaInP micro-LED without passivation were shown in Fig. 1b depending on the device sizes from 15 × 15 μm^2^ to 80 × 80 μm^2^. At the reverse bias region, the dark current densities were measured nearby the measurement floor, while it was clearly noted that there is a low leakage current that originates from parasitic current paths such as a sidewall [15]. Comparing the 15 × 15 μm^2^ device characteristics with the same sized device of Oh et al. our IV plot shows low leakage current and dark current density level. It suggested that there was negligible degradation through various fabrication processes. Also, the systematic current density increase over 2 V bias is found as the device size decreases. In Fig. 1c, the *J*–*L* (Current density–Light output power) characteristics of 20 × 20 μm^2^ are shown by comparing the same size device of Wong et al. [10]. Compared to the device of Wong et al. the 20 μm micro-LED of this paper showed 2.32 μW, which is 46 times higher at 20 A/cm^2^. And 6.0 μW which is 54 times at 40 A/cm^2^. They passivated the devices by depositing Al_2_O_3_ followed by nitrogen plasma surface treatment. Even comparing with the passivated micro-LED, our micro-LED showed 8 times higher at 20 A/cm^2^ and 8 times higher at 40 A/cm^2^. As a result, our micro-LED device showed a much larger light emission. The inset of Fig. 1d shows the microscope image with 100 A/cm^2^ current density injected to micro-LEDs. When the current of 100 A/cm^2^ is driven, the red light emission to the top surface of the 20 × 20 μm^2^ device is clearly shown. As shown in Fig. 1d, the electroluminescence (EL) spectra of 15 × 15 μm^2^ LEDs are shown with the peak wavelength 628 nm with current densities in ranges from 53 to 400 A/cm^2^. Resulting full-width at half maximum (FWHM) value at 100 A/cm^2^ is 14 nm, which is similar to previous reported LEDs [16]. Furthermore, it was found that the light intensity increases with an increase of the injection current density, and without the severe peak wavelength change. From these results, the fabricated micro-LEDs are well-functioned with our fabrication process.

**Fig. 2:**
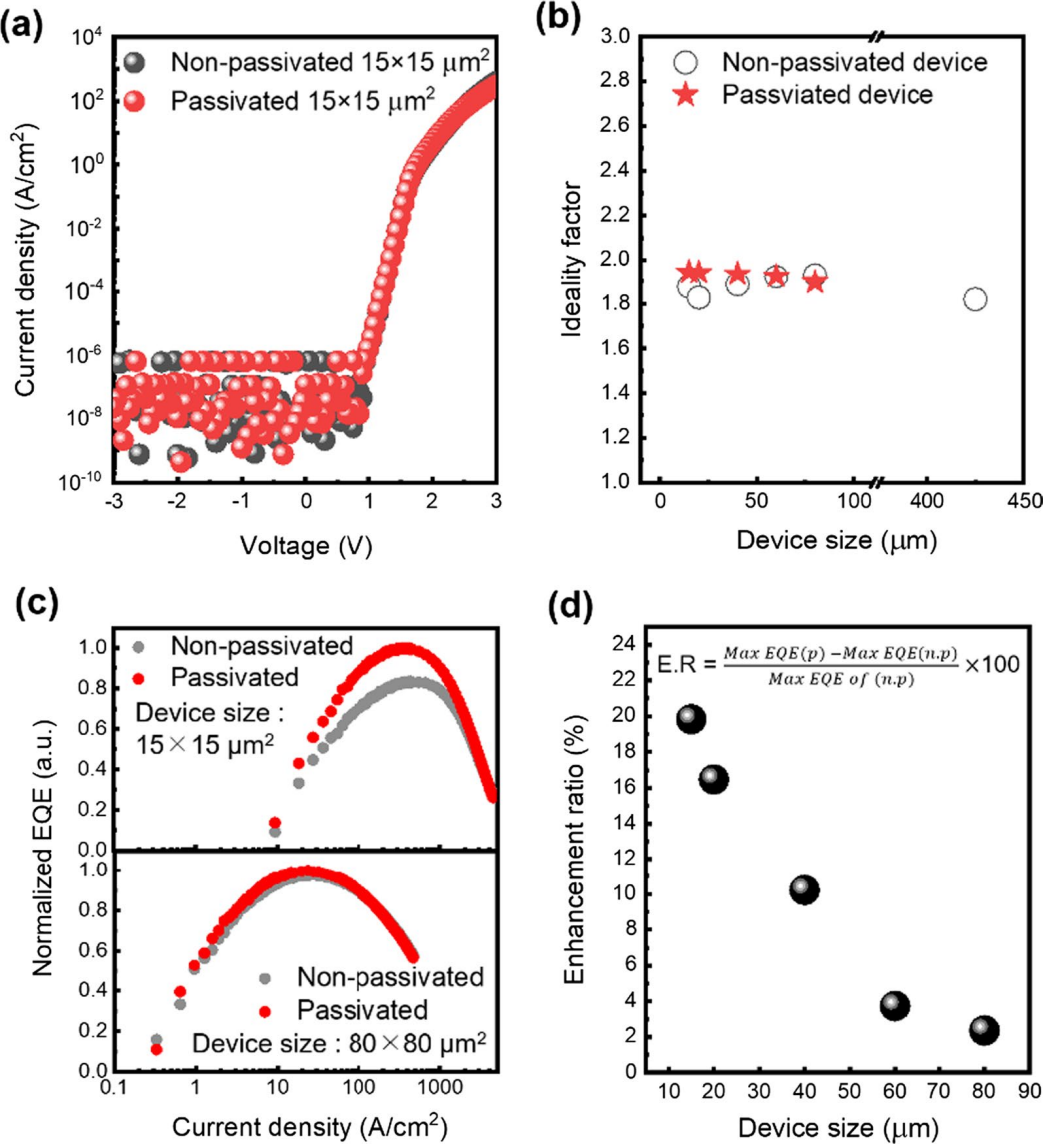
**a** *J–V* curves of non-passivated and passivated 15 × 15 μm^2^ LED. **b** Diode ideality factor depending on device sizes. **c** The EQE comparison of AlGaInP/GaInP LEDs which have the size of 15 × 15 μm^2^ and 80 × 80 μm^2^ with and without passivation. **d** The enhancement ratio of maximum EQE with passivation depending on the device sizes

To examine the passivation effects on the electrical characteristics, current density–voltage (*J*–*V*) measurements were conducted for non-passivated and passivated micro-LEDs with 15 × 15 μm^2^. In Fig. 2a, there were almost the same current density–voltage curves at the whole bias range. For other device sizes, it was noted that there were only negligible changes (not shown here). Compared with other reports having relatively high leakage current, despite passivation, the reduction of leakage current was not noticeable in our micro-LEDs [7, 10, 11]. Specifically, in Figs. 1b and 2a in our study, the leakage current of reverse bias was saturated from 10^−9^ to 10^−7^ A/cm^2^ in all device dimensions. The leakage current showed a very low level and the saturation behavior was very stable with increasing reverse bias. Additionally, in Figs. 1b and 2a, the *J*–*V* characteristics were shown in the log scale. Therefore, we can check there is no additional leakage current path nearby the threshold voltage at forward bias. These results strongly suggest that our devices inherently showed low leakage current level at both reverse and forward bias, thereby, the negligible variation for electrical properties was observed even after the surface passivation rather than other reports. It could be attributed to the inherently low leakage current with well-defined fabrication process, which could lead to very small deviation of leakage currents.

For the in-depth analysis, the diode ideality factor was calculated by Eq. (1)

1 $$n = \frac{q}{kT}\left( {\frac{\partial \ln I}{{\partial V}}} \right)^{ - 1}$$

where *n*, *q*, *k*, *T*, *I*, and *V* are the ideality factor, the elementary charge, the Boltzman constant, the absolute temperature, current, and the applied voltage, respectively. Figure 2b illustrates the ideality factors of non-passivated and passivated micro-LEDs depending on the device sizes from 15 × 15 μm^2^ to 100 × 100 μm^2^. The value of the 425 × 425 μm^2^ LED without passivation was also added for a large-size LED. Referring to the conventional interpretation of ideality factor in micro-LEDs, the ideality factor near unity means a radiative recombination dominance, while the ideality factor of 2 indicates SRH recombination dominance through trap states [17]. As depicted in Fig. 2b, the value of the ideality factor of the non-passivated device is from 1.82 (425 × 425 μm^2^) to 1.93 (80 × 80 μm^2^). The severe degradation of ideality factors depending on mesa sizes is not found compared to previous reports. Additionally, ideality factors of the passivated micro-LEDs are from 1.89 (80 × 80 μm^2^) to 1.94 (15 × 15 μm^2^), which are very similar to non-passivated LEDs. When compared to other reports, the noticeable increase of ideality factors was not observed with a reduction of micro-LED sizes and passivation. Although the ideality factor has been used as a good indirect indication for electrical characteristics of LED, it was not so effective to analyze the fabricated LEDs in this work. The difference of ideality factors depending on the device sizes are subtle like within 2 decimal places, thus it was quite difficult to evaluate the sidewall degradation or passivation effects from these values. Because our micro-LED devices showed an inherently low leakage current, which is close to measurement limits, the minor change in electrical characteristics with or without passivation couldn’t be measured.

In the fabrication process, the etching process using BOE and HCl was included. The buffered oxide etchant (BOE) and HCl have been reported as a good remover of the native oxide at the surface of the semiconductor. Thus, the electrical characteristics may look better immediately after immersion in the solutions. However, if there is no additional treatment like sulfur immersion, the sidewall will be gradually re-oxidized after enough time passes [18]. Because the micro-LEDs without passivation in the manuscript are measured after being exposed to air quite a few hours later, it doesn’t seem the wet treatments critically change the electrical measurement. Especially, HCl has been widely utilized as an etchant for AlGaInP epilayers or GaInP etch stop layer combining with H_3_PO_4_ when fabricating the red-LEDs [19–21]. However, those studies have not shown special electrical characteristics or high external quantum efficiency like this paper. This indirectly indicates that the usage of the chemicals such as H_3_PO_4_, HCl, BOE is not the main factor influencing the micro-LED performance. Also, all wet processes were equally addressed to each sample, thus, the impact of the passivation steps was fairly compared and the improvement by passivation steps is reasonable.

Figure 2c shows the normalized EQEs with and without passivation for 15 × 15 μm^2^ and 80 × 80 μm^2^ micro-LEDs. The values of EQE are normalized by the maximum values of passivated micro-LEDs for each size. The enhancement of the peak EQE values was clearly observed in the optical measurement unlike the electrical characteristics. Even though the electrical characteristics didn’t show definite difference because of the low leakage current level, the optical performance showed clear improvement by utilizing the passivation process.

To compare the effects of surface passivation depending on a different size, the enhancement ratio is defined by Eq. (2).

2 $$E{\text{nhancement ratio}} = \frac{{{\text{Max}}{\text{. EQE}} \left( {{\text{passivated}}} \right) - {\text{Max}}{\text{. EQE}} \left( {\text{non passivated}} \right)}}{{{\text{Max}}{\text{. EQE}} \left( {\text{non passivated}} \right)}}$$

The surface passivation increased the maximum EQE of 15 × 15 μm^2^ micro-LED as 19.8% and the maximum EQE of 80 × 80 μm^2^ as a 2.4%. Because of the higher surface-to-volume ratio, 15 × 15 μm^2^ was more affected by surface recombination of sidewall defects, so the passivation effect was larger than 80 × 80 μm^2^. Besides, calculated enhancement ratios for the different sizes is shown in Fig. 2d. The 60 μm and 80 μm micro-LEDs showed an enhancement ratio of less than 5%, but the device pitch smaller than 40 μm had a higher value over 10%. The enhancement ratio starts to increase rapidly under the device size of 40 μm. The large enhancement of EQEs in the smaller size micro-LEDs strongly suggests that our passivation could suppress the surface recombination for the AlGaInP/GaInP micro-LEDs smaller than 40 μm size. On the other hand, the EQE enhancement might be attributed to the increased LEE. Due to the lower refractive index of Al_2_O_3_, Al_2_O_3_ deposition decreases the difference of refractive index between LED epilayers and air. Therefore, less amount of photons would be guided in the epi-layers due to less total internal reflection. The LEE enhancement effect and the passivation effect seem to be mixed in the results. To clarify this issue, we simulated 40 μm micro-red-LED (GaP/Al(Ga)InP/MQW/Al(Ga)InP/GaAs substrate) by finite-difference time-domain (FDTD) simulation of LUMERICAL. The dipole source with the wavelength 625 nm is positioned in the center of MQWs and the transmittance towards the top and sidewall is calculated when 10 nm Al_2_O_3_ is deposited or not. As a result, the device with Al_2_O_3_ had a 1% decrease in top transmittance and no difference at the sidewall, which means if the thickness is not optimized, there is no LEE enhancement. Therefore, the EQE improvements of this work are not because of enhanced LEE by Al_2_O_3_ deposition but because of the reduced SRH recombination by passivation process.

Although the EQE enhancements were observed, through a simple optical characterization, we could relatively evaluate the effects of the passivation such as enhancement ratios. Thus, to compare the sidewall contribution of micro-LEDs, size-dependent electrical characterization, other than *J*–*V* curves or the ideality factor, and optical characterization should be simultaneously considered.

### The Extraction of a Surface Recombination Velocity for Micro-LEDs

To quantitatively confirm the sidewall contribution, it is necessary to evaluate the passivation effect not just by the simple electrical characteristics but by *J*_EQE, peak_ analysis with optical measurement. *J*_EQE, peak_ can be a useful analytical tool to analyze the impact of SRH recombination. Because *J*_EQE, peak_ is proportional to $\sqrt {A/C}$, in which *A* is the SRH recombination coefficient and *C* is the auger recombination coefficient respectively [22].

**Fig. 3:**
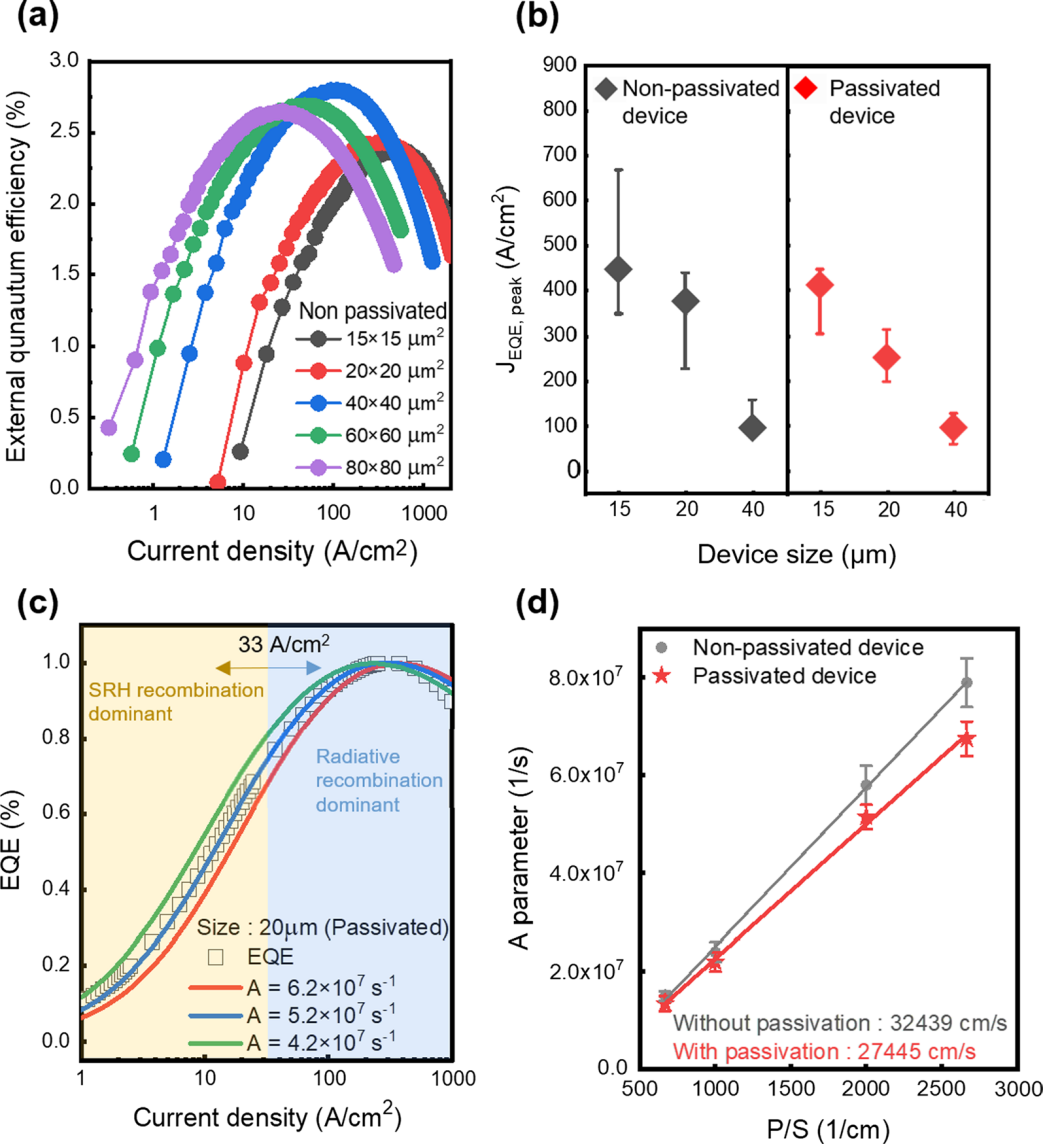
**a** Size-dependent EQE of AlGaInP/GaInP micro-LED. **b** Peak current density of LED devices (symbol) and the current density range 1% apart from peak current density (line) with (left) and without passivation (right). **c** The EQE plot fitting based on the ABC model. **d** Size-dependent SRH parameters were used in fitting the EQE curves with and without passivation. The surface recombination velocity is extracted from the slope

In Fig. 3a, the EQE of micro-LED without passivation is shown as a function of the current density with various sizes. In the literature, when the sidewall degradation has a significant impact on micro-LED, the smaller devices have lower EQE because of higher sidewall degradation [7]. However, in Fig. 3a the peak EQE is interestingly increased when the size is varied from 80 to 40 μm, and the peak EQE is decreased with a decrease the size from 40 to 15 μm. These results indicate a relatively lower impact of sidewall degradation than other reports due to the well-designed fabrication process and the impact of increased light extraction efficiency (LEE).

As the mesa size is decreased, the EQE curve shifts to a higher current density region, and *J*_EQE, peak_ becomes higher. The 15 × 15 μm^2^ device showed *J*_EQE, peak_ at 444 A/cm^2^, whereas relatively large 80 × 80 μm^2^ device showed 27 A/cm^2^. It shows a very significant feature of the degradation associated with the size scaling of the LED, indicating that the analysis of *J*_EQE, peak_ would be a key to capture the impact of the surface state at the sidewall.

In Fig. 3b, the bar plot shows the range of current density which has the EQE value within 1% deviation at the maximum point. The diamond symbol points to *J*_EQE, peak_. Dry etched sidewall surface could have a non-uniform surface condition in terms of defect density (damage). Therefore, before the annihilation of the defective region by the surface passivation, this non-uniformity might contribute to the variation of the *J*_EQE, peak_, as we measured. Therefore, we indicated *J*_EQE, peak_ value with error bar. The black and red plots represent the data without passivation and with passivation for each. Comparing the two plots, the *J*_EQE, peak_ shift was reduced with the passivation process. *J*_EQE, peak_ of the 15 × 15 μm^2^ device without the surface passivation ranges from 347 to 666 A/cm^2^. With the surface passivation, the range is lowered from 302 to 444 A/cm^2^. On the other hand, the 40 × 40 μm^2^ device is relatively less affected than a 15 × 15 μm^2^ device by sidewall recombination. Furthermore, the 60 × 60 μm^2^ and 80 × 80 μm^2^ size devices showed a slightly lowered peak current density, but the effect was negligibly small. Therefore, we can conclude that the sidewall contribution of AlGaInP/GaInP micro-LED is very critical under the size of 40 × 40 μm^2^, which is the dimension of our interest for the display to achieve high EQE and maintain peak current density. Also, it can be effectively reduced through wet treatment followed by depositing the Al_2_O_3_ passivation layer.

To deeply investigate the origin, analyzing the efficiency behavior by fitting through the ABC model is a useful strategy. The A, B, and C constant represents the SRH non-radiative recombination, the radiative recombination, and the Auger recombination for each. In the ABC model, the external quantum efficiency is expressed as follows [22].

3 $$\eta_{{{\text{ext}}}} = \eta_{{{\text{lee}}}} \eta_{{{\text{int}}}} ,\quad \eta_{{{\text{int}}}} = \frac{{\eta_{{{\text{inj}}}} Bn^{2} }}{{An + Bn^{2} + Cn^{3} }},$$

4 $$J = qw\left( {An + Bn^{2} + Cn^{3} } \right)$$

5 $$A = A_{0} + v_{{\text{s}}} \frac{P}{S}$$

Here, $\eta_{{{\text{ext}}}}$, $\eta_{{{\text{lee}}}}$, $\eta_{{\text{int}}}$, $\eta_{{{\text{inj}}}}$, and *n* are the external quantum efficiency, the light extraction efficiency (LEE), the internal quantum efficiency, the injection efficiency of the LED, and the carrier concentration, respectively. The ABC model assumes that the A, B, C constants and $\eta_{{{\text{lee}}}}$ of the LED chip are independent of the carrier concentration and operating current. The $\eta_{{{\text{lee}}}}$ is affected by factors that can influence the optical path of emitted photons such as device structures and layer thickness. Those factors don’t change when the miro-LEDs are operated with different current injection. Therefore, the LEE can be considered as constant. Also, the $\eta_{{{\text{inj}}}}$ is deserved as 100% at the low current density region in the MQW [23]. Therefore, considering constant $\eta_{{{\text{lee}}}}$ and $\eta_{{{\text{inj}}}}$, the experimental normalized EQE plot can be fitted by the normalized $\eta_{{\text{int}}}$ in Eq. (3). The current density of the ABC model is calculated by Eq. (4). *J* and *w* are the current density and the width of the quantum well. For the w value, 6 nm was used referring to the thickness of the quantum well of the epitaxial structure used in this study. The carrier concentrations (*n*) were swept from 10^15^ to 10^19^ cm^−3^ to calculate EQE as a function of the current density on a log scale. Based on those equations, the normalized measured EQE was fitted by the calculated internal quantum efficiency of the ABC recombination model. As the most governing parameter, we varied the values of the A value to investigate the impact of the surface recombination. On the other hand, we fixed the B and C coefficient as 1.5 × 10^−10^ cm^3^ s^−1^ and 4 × 10^−29^ cm^6^ s^−1^ since those parameters have minor changes on fitting results of the target current density range (Additional file 2: Figure S1) [24, 25]. In Fig. 3c, the normalized EQE of 20 × 20 μm^2^ is fitted by different A values from 4.2 × 10^7^ s^−1^ to 6.2 × 10^7^ s^−1^. Increasing A parameter moves to the *J*_peak, EQE_ to higher current density, reflecting the increased SRH recombination at the sidewall. Therefore, we chose the reasonable A values of good fitting results which induce less than 5% fitting difference between experimental data and calculated result. The fitting difference is defined as the ratio (%) of the difference between normalized EQE and normalized IQE to normalized EQE. The best fitting result is plotted by 5.20 × 10^7^ s^−1^ of A parameter in Fig. 3c. Our target fitting region is the current density around *J*_EQE, peak,_ and under *J*_EQE, peak_. This region is where SRH recombination (A parameter) has a dominant impact on IQE calculation and the magnitude of sidewall degradation can be shown. At high current region, because there are other hindrances such as current crowding effect or high series resistance, it is hard to obtain perfectly matched fitting results. Therefore, we fitting process was focused on the low current density region including *J*_EQE, peak_ where LEDs show the inherent performances without additional effects. The yellow and blue shadowing region represents which recombination mechanism is dominant.

The constant *A* which is the SRH recombination coefficient can be re-written with the bulk SRH coefficient *A*_0,_ the surface recombination velocity (SRV) $v_{{\text{s}}}$, perimeter *P* and area *S* in Eq. (5). The bulk term is *A*_0_ which describes that the SRH recombination at the bulk epi-layers, and the perimeter term is $v_{{\text{s}}} \cdot \frac{P}{S}$ which describes the SRH recombination at the device surface. To focus on the quantitative value of *v*_s_, we utilized the A, B, C recombination coefficients as keys to connect the experimental data and SRH recombination coefficient. Therefore we can extract the SRV by calculating the slope of *A* parameter versus *P*/*S* ratio depending on the device size by Eq. (5). In Fig. 3d, the SRH recombination constant (*A*) of with and without passivation are shown with the error bars. The median values of each range showed the best-matched fitting results and they were used to calculate SRV. In the case of passivation, the value of 27,445 cm/s was 15% lower than that of 32,439 cm/s without passivation by extracting the slopes. Although the extracted SRV values were not be decreased significantly, it is worth noting that the change of *J*_EQE, peak_ shift depending on the various micro-LED size allows us to extract the SRV values. Although the extracted SRV values were not be decreased significantly, it is worth noting that the change of *J*_EQE, peak_ shift depending on the various micro-LED size allows us to extract the SRV values.

Consequently, even if we achieved the peak EQE enhancement, the lower *J*_EQE, peak_, and the SRV reduction, the enhancement of device performance was not so dramatic, indicating further study should be carefully designed and carried out to maximize the performance of scaled LEDs. The possible reason for limited enhancement in this work is the incomplete removal of the defects at the sidewall. Since the sulfur treatment and the Al_2_O_3_ passivation are known to remove the native oxide of GaAs and reduce the surface density states near the conduction band edge [28], thereby, the surface trap states at the midgap and/or near the valence band can still be remained and limit the device performance enhancement even with the passivation. This suggests that further passivation study with a consideration of the amount of the trap along with the energy level will further improve the performance.

## Conclusion

In conclusion, we fabricated the AlGaInP/GaInP micro-LEDs and investigated the electrical and optical characteristics of the devices with the sizes from 15 × 15 μm^2^ to 80 × 80 μm^2^. In this process, the specific EQE value of the Red LED pixel was investigated to a low current density range and this data will be an important reference aimed at using the AlGaInP/GaInP red LED as a unit pixel micro-LED display. Also, the investigation on the effect of surface passivation with sulfur treatment followed by Al_2_O_3_ deposition was conducted. Since our micro-LEDs showed the inherently low leakage current before passivation with well-arranged fabrication process, there was no notable electrical difference with and without passivation. On the other hand, the clear EQE improvement and lowered *J*_EQE, peak_ was observed particularly in micro-LED with a size under 40 × 40 μm^2^. This motivated us to suggest a new analysis method to provide an insight into the surface state of the LEDs. Therefore, we first introduced the novel analysis methodology with *J*_EQE, peak_ to evaluate the sidewall effect in micro-LED. Additionally, we fitted the experimental EQE with the ABC recombination model and extracted the SRV from the size-dependent SRH constant (*A*), and demonstrated a 14% improvement with the passivation. The evaluation process shown in this paper can be used as a straightforward, but simple guideline to analyze the sidewall-related performance degradation or improvement of micro-LED. Consequently, to realize high EQE red micro-LED, further studies that can remove the trap sites at midgap and/or near the valence band should be carried out in the future.

## Supplementary Information

**Additional file 1:** Supplementary information for optical measurement for low current density: The photodetector measurement.

**Additional file 2:** Supplementary information for selection fitting parameter.

## Data Availability

The datasets used and analysed during the current study are available from the corresponding author on reasonable request.

## Abbreviations

Micro-LED: Micro-light-emitting-diode
EQE: External quantum efficiency
SRV: Surface recombination velocity
ICP-RIE: Inductively-coupled plasma reactive ion etching
SRH: Shockley–Read–Hall
ALD: Atomic layer deposition
MQW: Multi-quantum well
MOCVD: Metal–organic chemical vapor deposition
DI: Deionized
EL: Electroluminescence
FWHM: Full with at half maximum
BOE: Buffered oxide etchant
FDTD: Finite-difference time-domain
LEE: Light extraction efficiency
