# Supplementary material for: Understanding the Sidewall Passivation Effects in AlGaInP/GaInP Micro-LED
Source: Nanoscale Res Lett. 2022 Mar 1;17:29. doi: 10.1186/s11671-022-03669-5 (PMC8888782; doi:10.1186/s11671-022-03669-5)
Supplement: Supplementary file 1 — Additional file 1: Supplementary information for optical measurement for low current density: The photodetector measurement. [file 11671_2022_3669_MOESM1_ESM.docx]

**Supplementary information**

**[Supplementary information for optical measurement for low current density: The photodetector measurement.]**

We measured the optical characteristics of micro-LEDs by using two methods with photodetector or the integrating sphere. The integrated sphere system is suitable for LED devices for a large area. Because a considerable amount of photons is required for optical measurement due to the large distance between the LED and the photodetector and the additional loss within the integrating sphere. Therefore, the measurable current density range (or light intensity range) is more limited. To overcome this limitation and measure optical characteristics, we built another setup that can measure the light at a lower current density of micro-LED and it consists of only the photodetector located directly above the LED without the integrated sphere.

$$\text{Light output power }\left[ \text{W} \right]\text{=Photocurrent }\left[ \text{A} \right]\text{×Photo sensitivity }\left[ \text{W/A} \right]\text{ }\text{ }\left( \text{1} \right)$$

$$\text{Photosensitivity }\left[ \text{W/A} \right]\text{= }\frac{\text{Light output power from integrated sphere [W]}}{\text{Photocurrent }\left[ \text{A} \right]}\text{ }\text{ (2)}$$

The output result of the photodetector measurement is photocurrent (A). In order to get the external quantum efficiency, the measured photocurrent is necessary to be converted into the light output power (W). And to convert the photocurrent into the light output power (W), the photosensitivity (W/A) should be calculated as shown in Equation 1. Therefore, we compare the photocurrent (A) measured from the photodetector with the light output power (W) measured from the integrated sphere at each same current density and get the photosensitivity (W/A) as shown in Equation 2. Finally, after multiplying the photosensitivity by the photocurrent, the calculated light output power from the photodetector can be used to get EQE. Due to the very short distance between the micro-LED and photodetector and no loss of integrated sphere, the very small light output power (< 0.3 μW) at a low current density region is well-measured.

Figure S1. The EQE of $\text{20×20 }\text{μm}^{\text{2}}$measured by the photodetector and integrated sphere.

The plot shown above is a comparison of EQE obtained through the photodetectors and EQE obtained through the integrated sphere when the device size is $\text{20×20}\text{ }\text{μm}^{\text{2}}$. Because the two graphs match very well with little difference, we can confirm the photodetector measurement is reliable.

Consequently, the experimental data in Fig. 3(c) is measured by a setup with only the photodetector, and Fig. 3(a) is measured by the integrated sphere measurement system. Therefore, in Fig. 3(a) the EQE tail of each device was not measured due to the optical measurement limit of the system. Because the photodetector measurement consumes a lot of time and extra work, we used the integrated sphere measurement after confirming the coincidence of two equipment systems and good fitting results with a photodetector.
