# Supplementary material for: Understanding the Sidewall Passivation Effects in AlGaInP/GaInP Micro-LED
Source: Nanoscale Res Lett. 2022 Mar 1;17:29. doi: 10.1186/s11671-022-03669-5 (PMC8888782; doi:10.1186/s11671-022-03669-5)
Supplement: Supplementary file 2 — Additional file 2: Supplementary information for selection fitting parameter. [file 11671_2022_3669_MOESM2_ESM.docx]

**Supplementary information.**

**[Supplementary information for selection fitting parameter]**


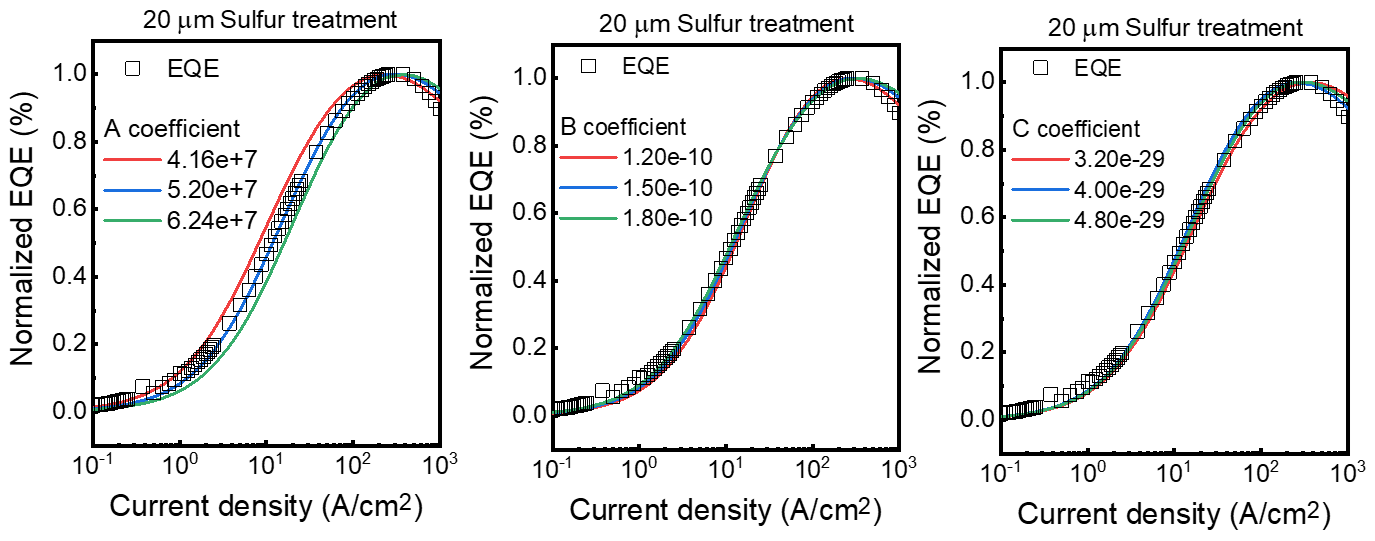


**Figure S1. Fitting results for 20**$\text{μm}$ **sulfur passivated micro-LED of changing A, B, and C fitting parameters by 20 %**

The three graphs above show the fitting results of changing A, B, and C by 20% alternatively with fixing the rest of the parameters. As we can see, changing the A parameter from 4.16×10^7^ s^-1^ to 6.24×10^7^ s^-1^ shows the largest deviation at the current density range less than J_EQE, peak_ and it changes the position of J_EQE, peak_. The EQE at this current density region is mainly dominated by the SRH recombination due to sidewall as shadows in Fig. 3(c) in the manuscript. The SRH recombination (A) is theoretically the only mechanism that induces the efficiency degradation by the sidewall status of the LED. The electronic states caused by the dangling bond or defects at the end of the crystal lattice are typically located within the bandgap and it serves as non-radiative recombination centers and contributes to the SRH recombination mechanism [E. F. Schubert, “Light-Emitting Diodes 2nd”, Cambridge University Press, Cambridge, New York, (2006)]. On the other hand, the radiative recombination (B) and the Auger recombination (C) less affect the IQE at low current density region and J_EQE, peak_, which are not the main interest in our discussion to investigate the impact of the surface on the quantum efficiency of the LED. Because the A parameter shows the greatest impact on the fitting results, especially at a low current density range including J_EQE, peak_, we chose it as a main changing value.
